# Radiosurgery for Symptomatic Cavernous Malformation in the Brainstem: Two Difficult Cases with Large and Multiple Lesions

**DOI:** 10.7759/cureus.6523

**Published:** 2019-12-31

**Authors:** Yoshihisa Kida

**Affiliations:** 1 Neurological Surgery, Ookuma Hospital, Nagoya, JPN

**Keywords:** cavernous malformation, brainstem, gamma knife

## Abstract

It is well known that cavernous malformations become much more hemorrhagic after the initial hemorrhage and that brainstem lesions are more dangerous than the lesions in the supratentorial location. It is very difficult to handle symptomatic cavernous malformations associated with repetitive hemorrhages in the brainstem. Patients may be suffering from brainstem syndromes such as hemiparesis, hemisensory disturbance, ataxia, and disturbed ocular movement. We have encountered two such difficult cases, one is very large and the other is multiple and familiar, accompanying repetitive brainstem hemorrhages. Since microsurgery seems to be very difficult and hazardous, these two cases were treated with radiosurgery after several hemorrhages.

## Introduction

Intracranial cavernous malformations (CMs) are congenital vascular anomalies similar to arteriovenous malformations, venous malformation, and capillary telangiectasia [1]. They are essentially very silent and benign disease and rarely become symptomatic. However, once they become symptomatic as a result of bleeding, seizures, or neurological deficits, they are often extremely troublesome due to repetitive episodes of bleeding and/or seizures, which are accompanied by neurological deterioration. Therefore, they often change their clinical course dramatically after the first incident. Several investigators have reported the so-called natural history of incidentally found CMs, and their reports have indicated a very favorable outcome [2-4]. However, the actual natural history of symptomatic CMs (s-CMs) after the first episode has not yet been confirmed and is likely very different from asymptomatic CMs [5]. Moreover, the lack of information regarding this natural history makes it difficult to clinically manage these patients, obscuring the value of currently used treatments such as surgery and radiosurgery. Since the brainstem CMs are believed to be much more hazardous, we will report the most troublesome cases in this location.

## Case presentation

Case 1

A 26-year-old male presented with no previous medical or surgical history. His illness started with occasional head heaviness over the right retromastoid portion. He complained of no weakness of the arm or leg, but sometimes complained of mild weakness of his left hand. He felt some difficulty in closing his eyes. After the initial complaint, he experienced headache several times and felt mild weakness and dysesthesia in his left arm and leg. When he experienced dysesthesia several times, he had to stop moving. No convulsive seizure occurred.

**Figure 1:**
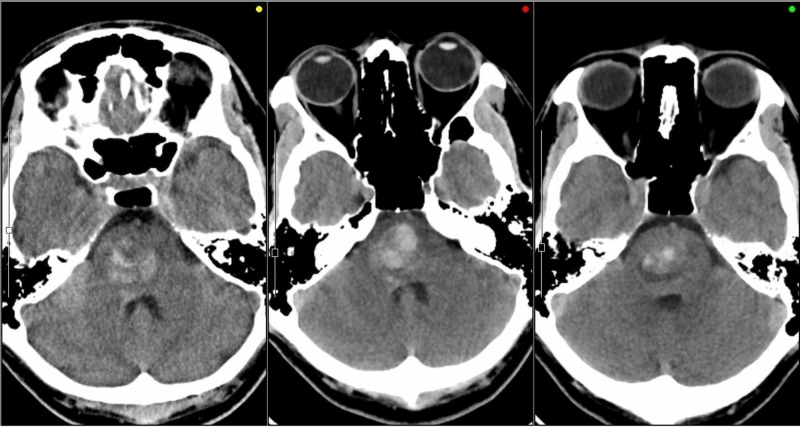
Serial CTs taken before Gamma Knife treatment An irregularly shaped high density was seen at the initial episode of headache (left), which apparently increased at the second episode two weeks later (center). Hematoma in the brainstem was rather decreased just before Gamma Knife treatment (right).

The first CT scan (Figure 1) showed a large high-density area in a heterogenous fashion in the center of the pons, indicating a minor hemorrhage. When he visited a nearby hospital due to headache, his CT scan showed an increased pontine hemorrhage. MRI showed a mixed high- and low-signal intensity lesion in the same area of the pons, demonstrating specifics of the CM associated with minor or moderate hemorrhage surrounded by expanding edema in the brainstem.

**Figure 2:**
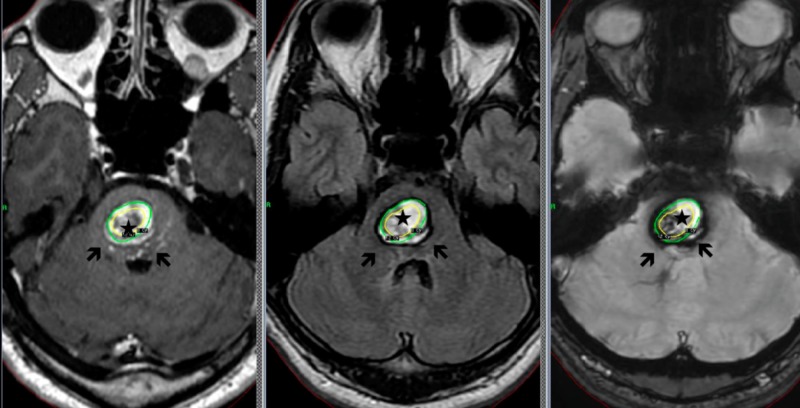
Cavernous malformation in the pons Only the core of cavernous malformation encircled with a yellow line (*) was irradiated, and the surrounding layer with hematomas (arrows) were out of target. Left: T1-weighted image; center: T2-weighted image; right: FLAIR (Fluid-attenuated inversion recovery) image

The core of CM encircled with a yellow line, not including the surrounding low-intensity rim, measuring 1 mL in volume was treated by Gamma Knife radiosurgery with a marginal dose of 12 Gy (Figure 2).

**Figure 3:**
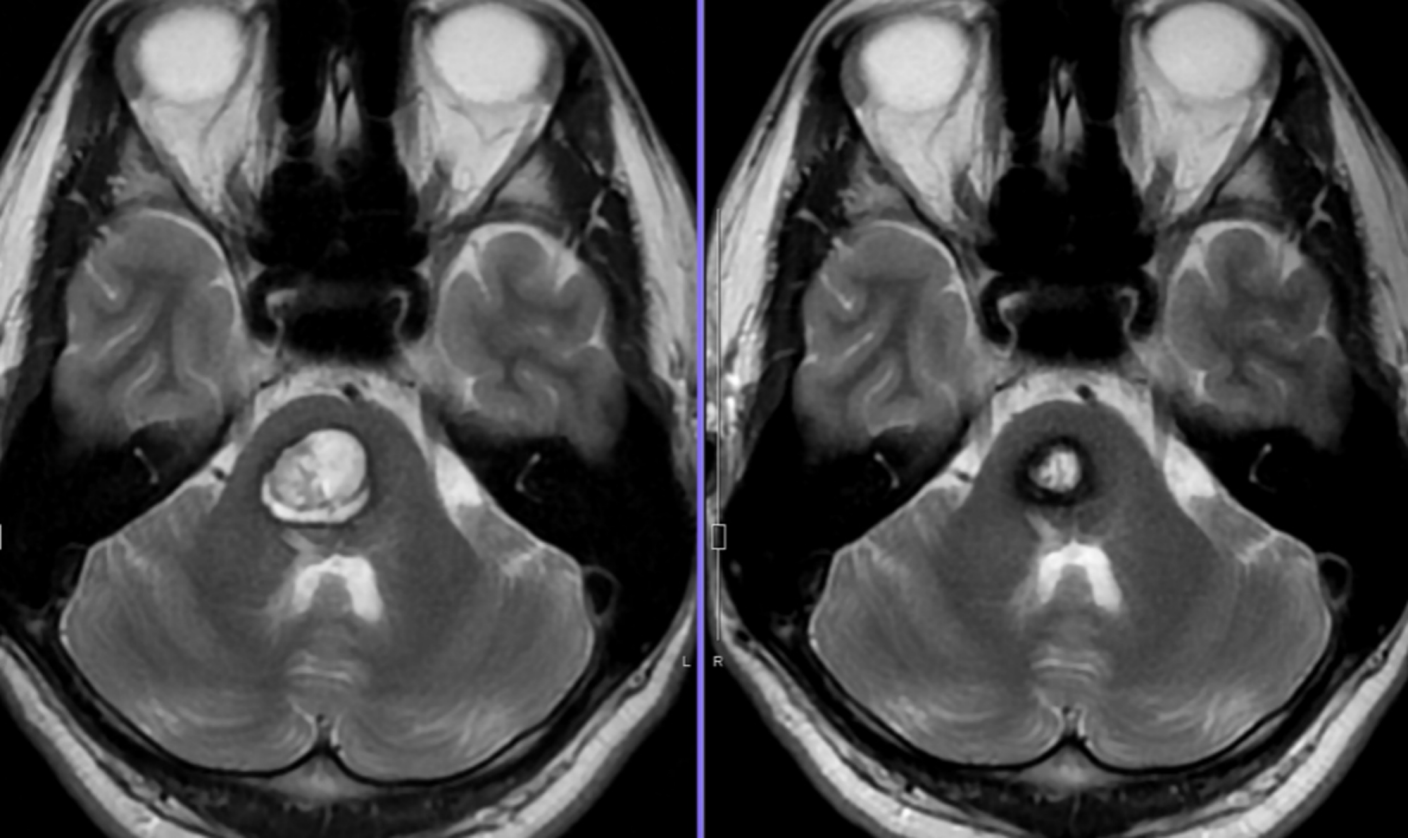
Follow-up MRI findings after radiosurgery A large lesion of cavernous malformation in the center of the brainstem (left) is much smaller in size six months later.

He experienced a fluctuating dysesthesia on the left half body for a while after the treatment, but no apparent weakness of the arm and leg occurred during nine months after Gamma Knife treatment. MRI showed no obvious changes in the CM, but showed a minor decrease in mass effects (Figure 3). He returned to daily work of business, and no incident occurred during the six months after radiosurgery.

Case 2

**Figure 4:**
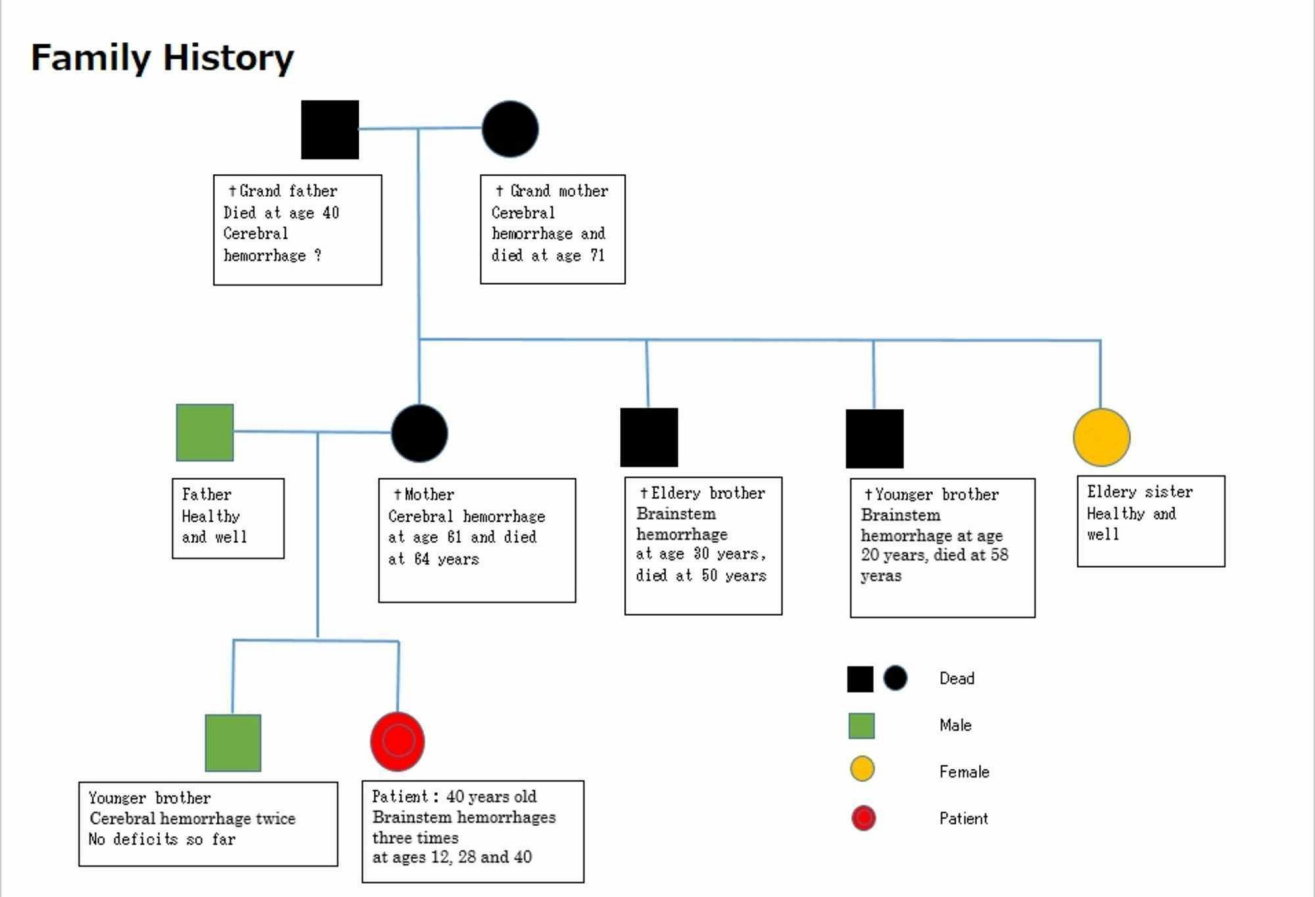
Family history of case 2 Many of her relatives were suffering from either cerebral or brainstem hemorrhages, indicating an apparently familiar and hereditary background.

A 41-year-old woman presented to our hospital seeking preventive methods of further bleeding subsequently after the last bleeding episode in the midbrain. She revealed that she had a sudden unconsciousness attack associated with nausea and vomiting at the age of 12 years. She was diagnosed as having brainstem hemorrhage due to CM, and multiple CM lesions in the entire brain. Her disturbed consciousness recovered within several months. She had an apparent hereditary background, and many of her relatives had been suffering from the same or similar disorders of the central nervous system (Figure 4).

**Figure 5:**
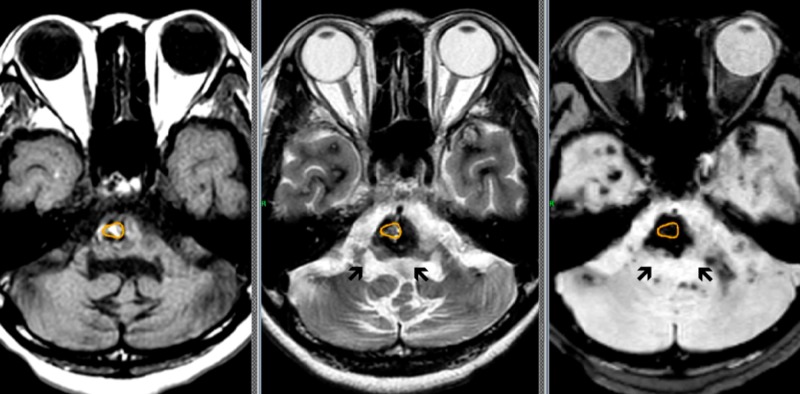
Multiple cavernous malformations in the brainstem Cavernous malformations (arrows) were treated by radiosurgery one by one and covered with a marginal dose of 11 Gy. Only the core of the lesions was covered as real targets (encircled). Many lesions of cavernous malformation were confirmed in the cerebellum and bilateral temporal lobe.

Thereafter, she had shown full recovery and had been well for the following 16 years. However, at the age of 28 years, she developed a second hemorrhage in the pons and a subsequent episode in the medulla. By the third attack and sudden onset of left hemiparesis and ophthalmoplegia, she was unable to walk. At this time, CT and MRI showed isolated hematoma in the left peduncle of midbrain. Several small CMs were confirmed in the supratentorial portion, which were not associated with an apparent hemorrhage (Figure 5).

**Figure 6:**
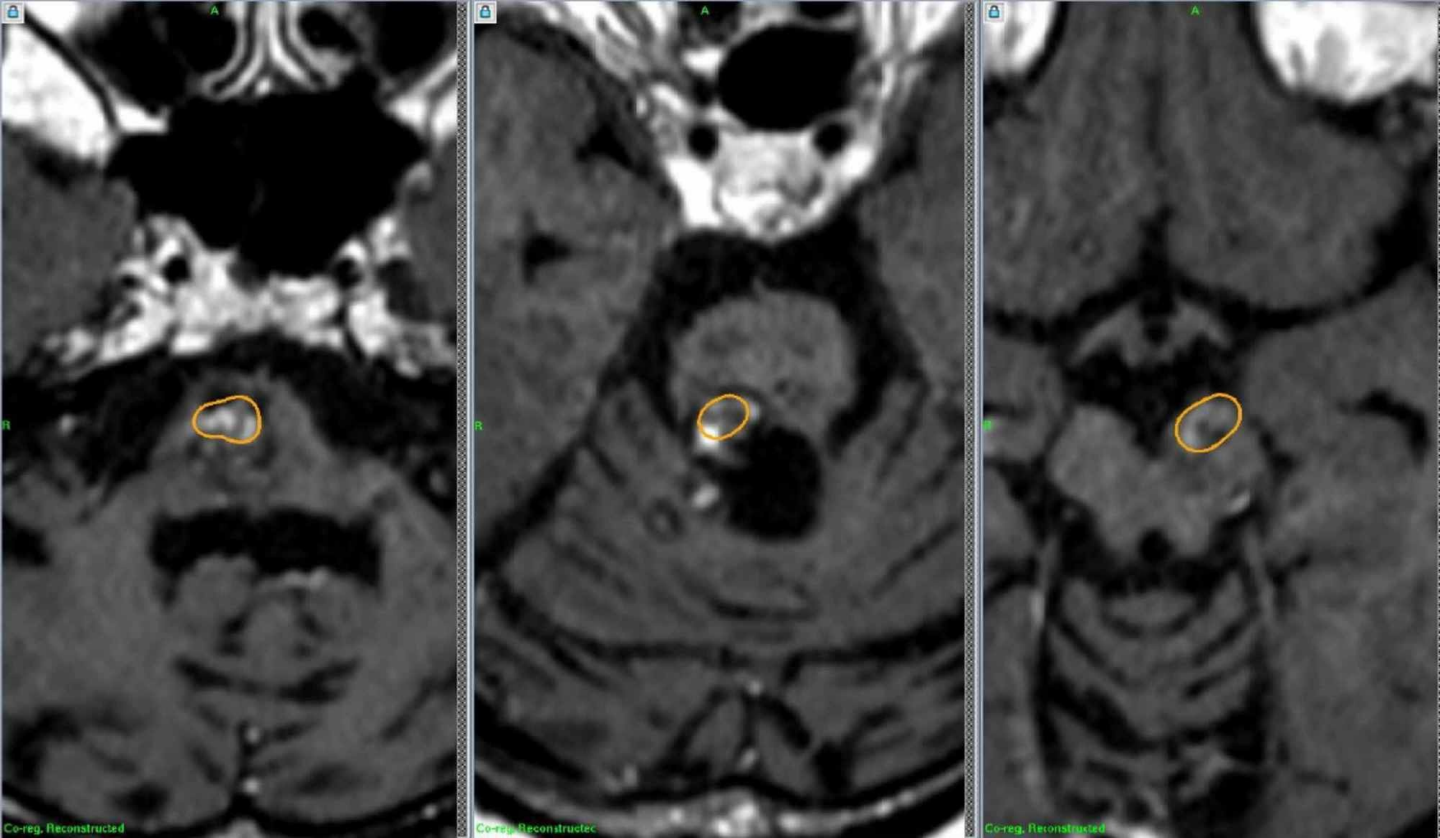
Follow-up MRIs after radiosurgery Coregistrated coronary MRI studies 12 months after radiosurgery showed no remarkable changes of the lesions in the medulla, pons, and midbrain,  when compared with the dose distribution at radiosurgery (encircled).

Since the CMs were in the pons, medulla, and midbrain, radiosurgery was performed one by one in two sessions, with the marginal doses of 11 Gy. After the radiosurgery, she has been well except for complex partial seizures associated with motor arrest and transient faintness, which occurred once or twice in a month. Follow-up MRIs after 12 months showed no further hemorrhage, and the lesions in the brainstem became smaller in size (Figure 6). She has been stable without showing perifocal edema at the 12 months after the treatment.

## Discussion

There has been a certain agreement that CMs rarely cause bleeding or seizure. Therefore, conservative treatment has been chosen for incidentally found CMs since the majority of them are believed to be asymptomatic overtime. Curling et al. [2] estimated the hemorrhage rate to be 0.25%/person-year/lesion, whereas Robinson et al. [4] reported a rate of 0.7%/person-year/lesion. However, symptomatic events of supratentorial lesions as well as brainstem lesions [3,5] may be altered tremendously after the first episode, and frequent hemorrhages or seizures may occur. Kondziolka et al. [5] reported that the bleeding rate was 0.6% per year without prior hemorrhage, 4.5% after a single hemorrhage, and 32% after two or more hemorrhages. Recently, there are a couple of reports showing a moderate increase or temporary clustering of hemorrhage after the first event [6-7], persistent at least for two to three years. It is often difficult to determine the definite timing when the asymptomatic lesions turn into symptomatic ones.

Surgical resection of s-CMs is apparently preferable to prevent further hemorrhages and intractable seizures. However, surgery is not always safe because CMs may originate in eloquent locations such as the brainstem or basal ganglia. Therefore, a selection bias for surgery is apparent, and only superficial as well as easily accessible CM lesions are currently operated. Nonetheless, several reports on surgical resection [8-9] are often accompanied by a lot of complications, especially in brainstem lesions. In recent reports on the surgery for brainstem CMs, favorable outcomes have been emphasized. Abla et al. [9] reported on the surgery of 260 cases of s-CM in the brainstem, in which perioperative complications and new deficits were 28% and 53%, respectively.

Radiosurgery with a Gamma Knife has been used in s-CMs in the brainstem as a treatment option. In the literature [10-15], successful results have been reported in terms of hemorrhage rate after the treatment. However, these results have always been controversial because of further hemorrhages and radiation-induced edema after the radiosurgery. There have been a lot of discussions to treat CMs with radiosurgery [16-17]. Moreover, the lack of information on the natural history of symptomatic lesions obscures the value of the treatment results. Although it is difficult to completely stop bleeding, radiosurgery seemingly reduces a further episode in many cases less invasively and with acceptable complications. However, overall results after radiosurgery have to be evaluated and compared with the real natural history of s-CMs, and whether or not radiosurgery can stop or improve the aggressive state of temporary clustering may hopefully be found by a randomized trial. In order to treat large and multiple brainstem lesions successfully, it is necessary to squeeze the target volume in the brainstem as less as possible and only the core of the CM lesions should be covered. For the multiple CMs in the brainstem, the treatments have to be staged or fractionated for the safety, as shown in our case. Because of hemosiderin deposition and its radio-sensitizing effects, the surrounding brainstem may have a danger of radiation injury. In our opinion, CMs in the brainstem should be treated with a dose of less than 15 Gy at the margin since the complication rates reportedly increased with the mean doses more than this dose [17].

## Conclusions

Two difficult cases of s-CM in the brainstem are reported, one is large and the other is multiple and familial. Both cases were not eligible candidates for microsurgery, and radiosurgery seemed to be a choice. It is crucial to shape up the target volume for large lesions, and only the core of CM should be treated with an adequate marginal dose of less than 13 Gy. On the contrary, it is important to select the dose and the timing either with a single session or multiple sessions when the lesions are multiple and adjacent to each other.
